# Factors related to sedentary behavior of pregnant women during the second/third trimester: prospective results from the large-scale Japan Environment and Children’s Study

**DOI:** 10.1186/s12889-024-20574-x

**Published:** 2024-11-15

**Authors:** Makie Nagai, Akiko Tsuchida, Kenta Matsumura, Haruka Kasamatsu, Hidekuni Inadera, the Japan Environment and Children’s Study Group

**Affiliations:** 1 Department of Public Health, Faculty of Medicine, University of Toyama, Toyama, Japan; 2 Toyama Regional Center for JECS, University of Toyama, Toyama, Japan

**Keywords:** Sedentary time, Screen time, Physical activity, Cohort study, Self-administered questionnaire

## Abstract

**Background:**

Prolonged sedentary behavior in pregnant women is reported to be associated with worse health-related indices and pregnancy outcomes. The aim of this study was to identify relevant factors that can be targeted in interventions to reduce sedentary behavior during pregnancy.

**Methods:**

Of 103,057 pregnancies registered in the Japan Environment and Children’s Study, 83,733 pregnant women were included for analysis after excluding multiple enrollments, nonresponses, and missing outcome data. Data were collected using the International Physical Activity Questionnaire and analyzed using logistic regression models to calculate crude and adjusted odds ratios. Missing data were handled using multiple imputations, and statistical analyses were performed using SAS software.

**Results:**

Mean sedentary behavior time increased from 5.4 h/day before pregnancy to 5.9 h/day during pregnancy. The percentage of women classified in the high sedentary behavior group increased from 25.6% before pregnancy to 31.2% during pregnancy. Factors associated with high sedentary behavior during pregnancy included longer hours spent watching television and playing video games before pregnancy, higher annual household income, and working status during pregnancy. Possible protective factors against high sedentary behavior included engaging in ≥ 150 min of moderate to vigorous physical activity per week before pregnancy.

**Conclusions:**

This large-scale cohort study provides valuable insights into sedentary behavior patterns among pregnant women in Japan. To reduce the amount of time engaged in sedentary behavior during pregnancy, the planning and management of time spent watching TV and playing video games as well as establishing exercise habits before pregnancy are recommended.

**Supplementary Information:**

The online version contains supplementary material available at 10.1186/s12889-024-20574-x.

## Background

Physical activity, including sedentary behavior (SB), during pregnancy is thought to affect the fetus by altering the intrauterine environment through energy metabolism and nutrient transport in the placenta. Too much time spent in SB during pregnancy is associated with high concentrations of C-reactive protein [1, 2] or low-density lipoprotein cholesterol [2], gestational diabetes mellitus [3, 4]. Furthermore, SB during pregnancy is also associated with the health of the fetus, including conditions such as inhibited fetal growth [5] and fetal macrosomia [6]. These findings suggest that SB can affect not only the health of the pregnant woman herself but also the health of her child.

Activities such as sitting, lying down, watching television (TV), and playing video games are defined as SB that does not significantly increase resting energy expenditure and correspond to 1.0–1.5 metabolic equivalent units (METs), which is close to the basal metabolic rate [7]. Although the World Health Organization recommends that pregnant women reduce the amount of time spent in SB and engage in physical activity instead [8], SB is known to increase during pregnancy compared with non-pregnancy [9, 10]. Even though an increase in SB during pregnancy may lead to poor outcomes, reducing SB in pregnant women is not easy. Therefore, it is necessary to identify factors that make it easier for pregnant women to reduce the amount of time spent in SB.

SB has been measured through self-administered questionnaires and wearable devices, but there is a high degree of heterogeneity in the literature [10]. Although there is some concern that studies have underestimated SB compared with instrumental measures [11], self-reported measures such as the International Physical Activity Questionnaire (IPAQ) [12] can be administered to a large target population, enabling the results to be compared internationally [13]. The aim of the present study is to identify how time spent engaged in SB changes in pregnant women before and during pregnancy by using IPAQ. We will also examine measures aimed at reducing SB during pregnancy by identifying the characteristics of the population with prolonged SB during pregnancy and associated factors.

## Methods

### Study design

The Japan Environment and Children’s Study (JECS) is an ongoing nationwide government-funded birth cohort study investigating the relationships between various environmental factors and children’s health and development. Details on the design and baseline characteristics of the JECS are available elsewhere [14, 15]. Briefly, expectant mothers were recruited between January 2011 and March 2014 at participating institutions in both rural and urban settings, including cooperating health care providers, across 15 regional centers throughout Japan. Data were collected using self-administered questionnaires or medical record transcriptions by physicians, midwives/nurses, and/or research coordinators.

The JECS protocol was reviewed and approved by the Ministry of Environment’s Institutional Review Board on Epidemiological Studies (100910001) and the Ethics Committees of all participating institutions. All participants provided written informed consent. Additionally, this study was approved by the Ethics Committee of University of Toyama (no. R2023172). All procedures in this study complied with the ethical standards of the relevant national and institutional committees on human experimentation and with the Code of Ethics of the World Medical Association (Declaration of Helsinki) for experiments involving humans.

### Study population

**Fig. 1:**
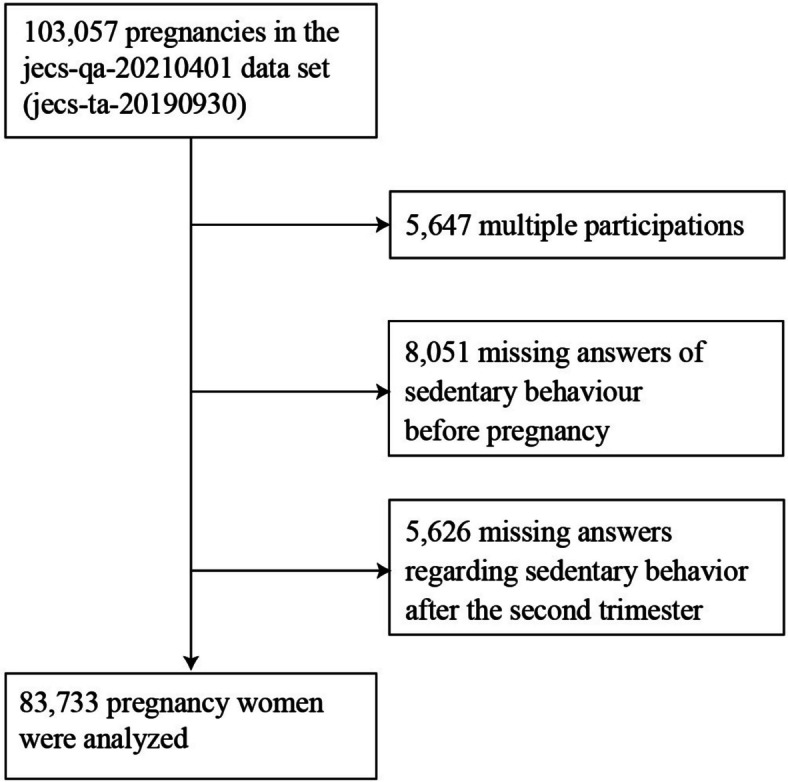
Participant flow diagram

The present study used the jecs-qa-20210401 dataset, which was released in April 2021, revised in February 2022, and contains data on 103,057 pregnant participants. After excluding 5,647 multiple participations (second or third registration of the same mother to the JECS), 8,051 missing answers regarding SB before pregnancy, and 5,626 missing answers regarding SB during pregnancy, a total of 83,733 participants were included in the final analysis (Fig. 1).

### Measures

#### Sedentary behavior

The number of hours spent engaged in SB on a weekday were measured using the Japanese version of the IPAQ short version [16]. The question was as follows: “How much time did you usually spend sitting/lying down on a weekday, including all the time spent sitting at a desk, talking with friends, reading, or sitting/lying down to watch television? Do not include sleeping time.” The response section of the questionnaire was in the form of “[$\cdot\cdot$] hours and [$\cdot\cdot$] minutes,” and participants responded with handwritten answers.

The questionnaire (M-T1) administered at the time of study registration (basically during the first trimester) included the phrase “think about the time you spent during an ordinary day before pregnancy.” Following this, the respondents were asked how much time they spent watching TV or playing video games during the entire time they were engaged in SB. Pre-pregnancy time spent watching TV and that spent playing video games were included in the analysis as independent variables of the subjects’ lifestyle. The questionnaire administered after the second trimester of pregnancy (M-T2) asked about time spent engaged in SB at the time of response. In this study, time spent engaged in SB during pregnancy was examined as an outcome.

The total number of hours spent engaged in SB was calculated using data from the dataset before any exclusions were made. In the calculations, responses that were not logical, such as answers exceeding 24 h or 60 min in the questionnaire forms, were excluded. Then, the 1st and 99th percentiles of time spent engaged in SB during pregnancy were calculated as 0 h/day and 17 h/day, respectively. Values of 0 h/day and over 17 h/day engaged in SB both before and during pregnancy were replaced with “missing” and were excluded from the analysis.

Referring to previous studies [17–19], we defined the “high-SB group” as women who engaged in SB ≥ 8 h/day and the “low-SB group” as those who engaged in SB < 8 h/day. Because there is no evidence to support a cutoff value for SB in pregnant women, 8 h was used in this study based on evidence from the general adult population in Japan.

#### Items considered for association

The following variables were selected to examine the associations with SB during pregnancy: number of hours watching TV before pregnancy, number of hours playing video games before pregnancy, number of hours sleeping during the first trimester, maternal age (< 25, 25–29, 30–34, or ≥ 35 years), annual household income (< 4, 4– < 6, or ≥ 6 million Japanese yen (JPY)), highest educational level (≤ 12, > 12– < 16, or ≥ 16 years), working status during the first trimester (unemployed or employed), parity (primipara or multipara), conception achieved through reproductive medicine (no/yes), multiple pregnancy (no/yes), pre-pregnant body mass index (BMI) (< 18.5, 18.5– < 25, or ≥ 25), type of residence (stand-alone house, living on the 5th floor or lower of a residential complex, living on the 6th floor or higher of a residential complex, other type of residence), smoking status (never smoked, quit before pregnancy, quit after pregnancy, or current smoker), alcohol intake (never consumed alcohol, quit, or current drinker); nausea and vomiting during pregnancy (NVP) prior to 12 gestational weeks (did not experience, nausea only, experienced NVP but could have meals, or experienced NVP and could not have meals); stressful events occurred after pregnancy (no/yes), psychological distress during the first trimester (total Kessler 6 (K6) score < 5, 5–12, and ≥ 13) [20], moderate-intensity physical activity (MVPA) per week calculated from the time written in IPAQ forms (< 150 min or ≥ 150 min) [8], dog ownership (no/yes), and physical and mental health (SF-8 score). Data on all items were obtained from self-administered questionnaires (M-T1 and M-T2) administered during pregnancy, while data on parity and BMI were collected from medical record transcripts.

### Statistical analysis

Descriptive characteristics are presented as percentages or as means and standard deviations (SD). Logistic regression models were used to calculate the crude and adjusted odds ratios (cOR and aOR) of becoming high-SB (≥ 8 h/day) in the second/third trimester of pregnancy and the corresponding 95% confidence intervals (CI). The low-SB and high-SB groups were stratified based on pre-pregnancy hours engaged in SB.

Missing data were treated using multiple imputations. The missing data rate was ≤ 1.0% for most variables, except for number of hours spent watching TV (7.6%), annual household income (6.3%), number of hours spent playing video games (3.9%), working status during the first trimester (3.1%), MVPA per week (2.9%), number of hours spent sleeping during the first trimester (2.5%), SF-8 scores for physical and mental health (1.4%), and multiple pregnancy (1.1%). Ten imputed datasets were created using chained equations [21], and the obtained results were combined using Rubin’s rule [22].

All statistical analyses were performed using SAS ver. 9.4 (SAS Institute Inc., Cary, NC).

## Results

**Fig. 2:**
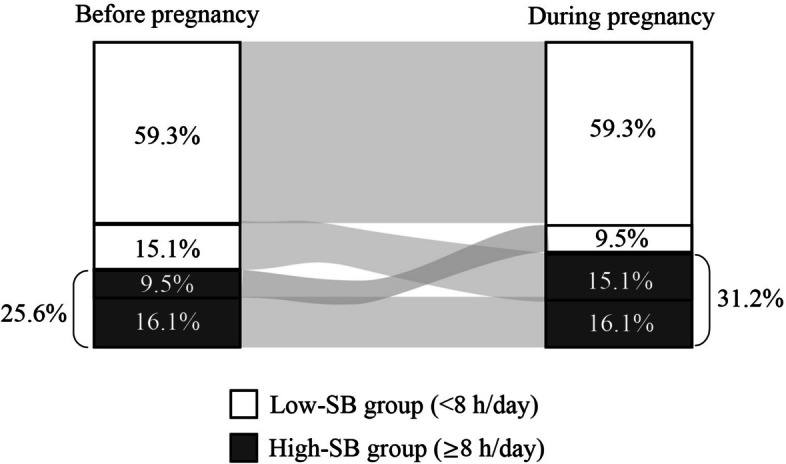
Percentages of women classified in the low- and high-SB groups before and during pregnancy

Data from 83,733 pregnant women were analyzed. Among these women, the average number of hours spent engaged in SB on weekdays before and during pregnancy was 5.4 (SD = 3.4) and 5.9 (SD = 3.5), respectively (Supplementary Fig. 1). The Pearson correlation coefficient for SB time before and during pregnancy was 0.478 (*p* < 0.0001). Before pregnancy, 25.6% of the women were classified in the high-SB group, which increased to 31.2% during pregnancy. Overall, 15.1% of participants changed from the low-SB group before pregnancy to the high-SB group during pregnancy, whereas 9.5% changed from the high-SB group before pregnancy to the low-SB group during pregnancy (Fig. 2).

Table 1 shows representative values for the items analyzed according to SB group before pregnancy. Sleep duration in the first trimester of pregnancy was almost the same for both groups. There was no significant difference in age composition, but higher percentages of the highest income group, highest education group, primiparas, and the group with less than the recommended level of MVPA were classified in the high-SB group.

**Table 1:** Participant characteristics according to amount of time spent engaged in sedentary behavior before pregnancy (*N* = 83,733)

	Sedentary behavior before pregnancy	
	< 8 h/day *n* = 62,349 (74.5%)	≥ 8 h/day *n* = 21,384 (25.5%)
**Duration of sedentary behavior before pregnancy**		
Hours, mean (SD)	3.8 (1.7)	10.3 (2.2)
**Duration of watching TV before pregnancy**		
Hours, mean (SD)	2.2 (1.3)	3.3 (2.2)
**Duration of playing video games**		
Hours, mean (SD)	0.4 (0.7)	0.6 (1.2)
**Duration of sleep during the first trimester**		
Hours, mean (SD)	7.5 (1.2)	7.4 (1.2)
**Age at baseline, n (%)**		
< 25	6,801 (10.9)	2,411 (11.3)
25–29	18,436 (29.6)	6,256 (29.3)
30–34	21,909 (35.1)	7,404 (34.6)
≥ 35	15,188 (24.4)	5,309 (24.8)
**Annual household income (JPY), n (%)**		
< 4 million	23,989 (41.1)	7,270 (36.2)
4– < 6 million	19,611 (33.6)	6,470 (32.2)
≥ 6 million	14,752 (25.3)	6,335 (31.6)
**Highest educational level (year), n (%)**		
< 13	22,024 (35.5)	7,636 (35.8)
13– < 16	27,507 (44.3)	7,695 (36.1)
≥ 16	12,590 (20.3)	5,988 (28.1)
**Working status, n (%)**		
Unemployed	23,378 (38.8)	6,616 (31.8)
Employed	36,924 (61.2)	14,196 (68.2)
**Parity, n (%)**		
Primipara	24,604 (39.8)	12,153 (57.3)
Multipara	37,292 (60.2)	9,054 (42.7)
**Conception achieved through reproductive medicine, n (%)**		
No	58,137 (93.7)	19,353 (90.9)
Yes	3,939 (6.3)	1,931 (9.1)
**Multiples pregnancy, n (%)**		
No	61,062 (99.0)	20,919 (99.0)
Yes	588 (1.0)	213 (1.0)
**BMI before pregnancy, n (%)**		
< 18.5	9,885 (15.9)	3,583 (16.8)
18.5– < 25	45,898 (73.6)	15,586 (72.9)
≥ 25	6,545 (10.5)	2,207 (10.3)
**Type of residence, n (%)**		
Stand-alone house	29,823 (48.2)	9,290 (43.8)
Residential complex ≤ 5th floor	25,085 (40.5)	9,293 (43.8)
Residential complex ≥ 6th floor	6,190 (10.0)	2,393 (11.3)
Other types of residence	782 (1.3)	239 (1.1)
**Smoking status, n (%)**		
Never smoked	35,839 (57.9)	13,104 (61.6)
Quit before pregnancy	14,690 (23.7)	4,642 (21.8)
Quit after pregnancy	8,455 (13.7)	2,625 (12.3)
Current smoker	2,933 (4.7)	899 (4.2)
**Alcohol intake during the first trimester, n (%)**		
Never consumed alcohol	21,557 (34.7)	7,036 (33.0)
Quit	34,357 (55.3)	11,983 (56.2)
Current drinker	6,190 (10.0)	2,299 (10.8)
**NVP prior to 12 gestational weeks, n (%)**		
Did not experience	10,311 (16.6)	4,035 (18.9)
Nausea only	26,591 (42.8)	9,225 (43.3)
Experienced NVP but could have meals	18,156 (29.2)	5,947 (27.9)
Experienced NVP and could not have meals	7,109 (11.4)	2,118 (9.9)
**Stressful events during pregnancy, n (%)**		
No	34,816 (56.2)	12,044 (56.7)
Yes	27,106 (43.8)	9,211 (43.3)
**Psychological distress during the first trimester (total K6 score), n (%)**		
< 5	42,186 (67.8)	14,575 (68.2)
5–12	17,887 (28.7)	6,091 (28.5)
≥ 13	2,188 (3.5)	693 (3.2)
**MVPA per week, n (%)**		
< 150 min	40,491 (67.1)	17,234 (82.2)
≥ 150 min	19,835 (32.9)	3,727 (17.8)
**Dog ownership, n (%)**		
No	53,970 (86.6)	18,654 (87.2)
Yes	8,379 (13.4)	2,730 (12.8)
**Physical and mental health (SF-8 score)**		
Physical component summary scores, mean (SD)	45.1 (7.3)	45.0 (7.4)
Mental component summary scores, mean (SD)	46.0 (7.2)	46.4 (7.2)

*BMI* body mass index, *JPY* Japanese yen, *K6* Kessler 6, *MVPA* moderate to vigorous physical activity, *NVP* nausea and vomiting in pregnancy, *SD* standard deviation, *TV* television

The cOR and aOR for the low- and high-SB groups during pregnancy were calculated for each item, and these were used to stratify the low and high-SB groups before pregnancy. Table 2 shows the results for those who were classified in the low-SB group before pregnancy. The results revealed that longer hours of playing video games before pregnancy, longer hours of watching TV before pregnancy, multiple pregnancy, living in a residential complex, alcohol intake during the first trimester, and the occurrence of stressful events during pregnancy were associated with high SB during pregnancy. In contrast, longer hours of sleep during the first trimester, multipara, higher annual household income, having 13– < 16 years of education, being employed during the first trimester, experiencing NVP and not being able to have meals, engaging in ≥ 150 min of MVPA per week, and higher SF-8 scores were associated with inhibition of high SB during pregnancy.

**Table 2:** Odds ratios (95% CIs) for ≥ 8 h/day of sedentary behavior after the second trimester among women who engaged in < 8 h/day of sedentary behavior before pregnancy (*N* = 62,349)

	Case ≥ 8 h/day	Subtotal	Prevalence	cOR(95% CI)	aOR^a^ (95% CI)
**Time spent of watching TV before pregnancy**					
By hour	-	-	-	**1.26 (1.25, 1.28)**	**1.20 (1.18, 1.22)**
**Time spent of playing video games before pregnancy**					
By hour	-	-	-	**1.23 (1.21, 1.26)**	**1.09 (1.07, 1.12)**
**Duration of sleep during the first trimester**					
By hour	-	-	-	**0.90 (0.88, 0.91)**	**0.97 (0.96, 0.99)**
**Age at baseline**					
< 25	1,728	6,803	25.4%	Reference	Reference
25–29	3,948	18,442	21.4%	**0.80 (0.75, 0.85)**	1.00 (0.94, 1.06)
30–34	4,165	21,914	19.0%	**0.69 (0.65, 0.73)**	1.01 (0.94, 1.08)
≥ 35	2,828	15,190	18.6%	**0.67 (0.63, 0.72)**	1.04 (0.97, 1.12)
**Annual household income (JPY)**					
< 4 million	5,609	25,941	21.6%	Reference	Reference
4– < 6 million	3,976	20,872	19.0%	**0.80 (0.77, 0.84)**	**0.90 (0.86, 0.95)**
≥ 6 million	3,083	15,536	19.8%	0.97 (0.92, 1.02)	**0.93 (0.88, 0.98)**
**Highest educational level (year)**					
< 13	4,863	22,118	22.0%	Reference	Reference
13– < 16	5,099	27,596	18.5%	**0.85 (0.81, 0.89)**	**0.82 (0.79, 0.86)**
≥ 16	2,706	12,635	21.4%	**0.90 (0.85, 0.94)**	1.03 (0.97, 1.09)
**Working status during the first trimester**					
Unemployed	4,703	24,368	19.3%	Reference	Reference
Employed	7,966	37,981	21.0%	**1.11 (1.06, 1.16)**	**0.91 (0.88, 0.96)**
**Parity**					
Primipara	7,334	24,932	29.4%	Reference	Reference
Multipara	5,334	37,417	14.3%	**0.40 (0.38, 0.42)**	**0.43 (0.41, 0.45)**
**Conception achieved through reproductive medicine**					
No	11,685	58,391	20.0%	Reference	Reference
Yes	983	3,958	24.8%	**1.32 (1.23, 1.42)**	1.06 (0.98, 1.14)
**Multiples pregnancy**					
No	12,466	61,749	20.2%	Reference	Reference
Yes	202	600	33.7%	**12.39 (6.70, 22.91)**	**1.87 (1.61, 2.18)**
**BMI before pregnancy**					
< 18.5	2,047	9,889	20.7%	1.03 (0.98, 1.09)	1.00 (0.94, 1.05)
18.5– < 25	9,283	45,914	20.2%	Reference	Reference
≥ 25	1,338	6,547	20.4%	1.01 (0.95, 1.08)	1.01 (0.94, 1.07)
**Residence, n (%)**					
Stand-alone house	5,389	30,041	17.9%	Reference	Reference
Residential complexes ≤ 5th floor	5,832	25,312	23.0%	**1.37 (1.31, 1.43)**	**1.10 (1.05, 1.15)**
Residential complexes ≥ 6th floor	1,285	6,208	20.7%	**1.19 (1.12, 1.28)**	**1.11 (1.03, 1.18)**
Other types of residence	162	788	20.6%	1.19 (0.995, 1.41)	0.98 (0.83, 1.17)
**Smoking during the first trimester**					
Never smoked	7,291	36,052	20.2%	Reference	Reference
Quit before pregnancy	2,792	14,799	18.9%	**0.92 (0.87, 0.96)**	0.96 (0.92, 1.01)
Quit after pregnancy	1,972	8,534	23.1%	**1.19 (1.12, 1.25)**	1.02 (0.96, 1.08)
Current smoker	612	2,964	20.6%	1.03 (0.94, 1.13)	1.04 (0.95, 1.15)
**Alcohol intake during the first trimester**					
Never consumed alcohol	4,137	21,646	19.1%	Reference	Reference
Quit before pregnancy	7,287	34,490	21.1%	**1.13 (1.09, 1.18)**	**1.07 (1.03, 1.12)**
Current drinker	1,245	6,213	20.0%	1.06 (0.99, 1.14)	**1.17 (1.09, 1.25)**
**NVP prior to 12 gestational weeks**					
Did not experience	2,225	10,342	21.5%	Reference	Reference
Nausea only	5,270	26,670	19.8%	**0.90 (0.85, 0.95)**	0.96 (0.91, 1.02)
Experienced NVP but could have meals	3,664	18,209	20.1%	**0.92 (0.87, 0.97)**	0.97 (0.91, 1.03)
Experienced NVP and could not have meals	1,510	7,129	21.2%	0.98 (0.91, 1.05)	**0.89 (0.82, 0.96)**
**Stressful events during pregnancy**					
No	7,010	35,046	20.0%	Reference	Reference
Yes	5,658	27,303	20.7%	**1.05 (1.01, 1.09)**	**1.05 (1.01, 1.09)**
**Psychological distress during the first trimester (total K6 score)**					
< 5	8,196	42,237	19.4%	Reference	Reference
5–12	3,919	17,922	21.9%	**1.16 (1.11, 1.21)**	0.99 (0.94, 1.04)
≥ 13	553	2,190	25.3%	**1.40 (1.28, 1.54)**	1.03 (0.93, 1.14)
**MVPA per week**					
< 150 min	8,603	41,265	20.8%	Reference	Reference
≥ 150 min	4,065	21,084	19.3%	**0.91 (0.87, 0.95)**	**0.94 (0.90, 0.98)**
**Dog ownership**					
No				Reference	Reference
Yes				0.99 (0.93, 1.04)	0.96 (0.91, 1.02)
**Physical and mental health (SF-8 score)**					
Physical component summary scores (by 1 point)	-	-	-	**0.98 (0.979, 0.984)**	**0.98 (0.979, 0.985)**
Mental component summary scores (by 1 point)	-	-	-	**0.99 (0.985, 0.990)**	**0.99 (0.990, 0.996)**

*aOR* adjusted odds ratio, *BMI* body mass index, *CI* confidence interval, *cOR* crude odds ratio, *JPY* Japanese yen, *K6* Kessler 6, *MVPA* moderate to vigorous physical activity, *NVP* nausea and vomiting in pregnancy, *TV* television

^a^Adjusted for each other; “—” denotes reference

Table 3 shows the results for those who were classified in the high-SB group before pregnancy. As in the pre-pregnancy low-SB group, longer time spent playing video games and longer time spent watching TV before pregnancy were associated with higher SB during pregnancy. The risk factors for the high-SB group during pregnancy that differed from the low-SB group described above were older age, higher annual household income, being employed during the first trimester, and BMI score before pregnancy < 18.5. The protective factors against being in the high-SB group during pregnancy that differed from the low-SB group described above were smoking experience, having a K6 score > 13 of and dog ownership.

**Table 3:** Odds ratios (95% CIs) for ≥ 8 h/day of sedentary behavior after the second trimester among women who engaged in ≥ 8 h/day of sedentary behavior before pregnancy (*N* = 21,384)

	Case ≥ 8 h/day	Subtotal	Prevalence	cOR(95% CI)	aOR^a^ (95% CI)
**Time spent of watching TV before pregnancy**					
By hour	-	-	-	**1.01 (1.0003, 1.03)**	**1.06 (1.05, 1.08)**
**Time spent of playing video games before pregnancy**					
By hour	-	-	-	0.99 (0.97, 1.02)	**1.05 (1.02, 1.08)**
**Duration of sleep the first trimester**					
By hour	-	-	-	**0.90 (0.88, 0.92)**	**0.96 (0.94, 0.99)**
**Age at baseline**					
< 25	1,393	2,411	57.8%	Reference	Reference
25–29	3,862	6,258	61.7%	**1.18 (1.07, 1.29)**	1.09 (0.99, 1.21)
30–34	4,753	7,405	64.2%	**1.31 (1.19, 1.44)**	**1.25 (1.12, 1.38)**
≥ 35	3,464	5,310	65.2%	**1.37 (1.24, 1.51)**	**1.29 (1.15, 1.44)**
**Annual household income (JPY)**					
< 4 million	4,611	7,876	58.5%	Reference	Reference
4– < 6 million	4,286	6,860	62.5%	**1.18 (1.10, 1.26)**	**1.10 (1.02, 1.18)**
≥ 6 million	4,575	6,648	68.8%	**1.56 (1.46, 1.68)**	**1.35 (1.24, 1.47)**
**Highest educational level (year)**					
< 13	4,703	7,662	61.4%	Reference	Reference
13– < 16	4,711	7,721	61.0%	0.98 (0.92, 1.05)	**0.84 (0.79, 0.90)**
≥ 16	4,058	6,001	67.6%	**1.31 (1.22, 1.41)**	1.00 (0.92, 1.08)
**Working status**					
Unemployed	3,971	6,905	57.5%	Reference	Reference
Employed	9,501	14,479	65.6%	**1.41 (1.33, 1.50)**	**1.38 (1.28, 1.48)**
**Parity**					
Primipara	8,325	12,296	67.7%	Reference	Reference
Multipara	5,147	9,088	56.6%	**0.62 (0.59, 0.66)**	**0.65 (0.61, 0.70)**
**Conception achieved through reproductive medicine**					
No	12,130	19,441	62.4%	Reference	Reference
Yes	1,343	1,943	69.1%	**1.35 (1.21, 1.50)**	1.05 (0.94, 1.18)
**Multiples pregnancy**					
No	13,323	21,165	62.9%	Reference	Reference
Yes	149	219	68.0%	1.25 (0.93, 1.69)	1.15 (0.85, 1.55)
**BMI before pregnancy**					
< 18.5	2,310	3,584	64.5%	1.07 (0.99, 1.15)	**1.09 (1.005, 1.17)**
18.5– < 25	9,805	15,591	62.9%	Reference	Reference
≥ 25	1,357	2,209	61.4%	0.94 (0.86, 1.03)	1.02 (0.93, 1.12)
**Residence, n (%)**					
Stand-alone house	5,798	9,371	61.9%	Reference	Reference
Residential complexes lower than ≤ 5th floor	5,968	9,367	63.7%	**1.08 (1.02, 1.15)**	1.00 (0.93, 1.06)
Residential complexes higher than ≥ 6th floor	1,570	2,403	65.3%	**1.16 (1.06, 1.28)**	1.01 (0.92, 1.11)
Other types of residence	136	243	56.0%	0.79 (0.61, 1.02)	0.81 (0.63, 1.06)
**Smoking during the first trimester**					
Never smoked	8,541	13,161	64.9%	Reference	Reference
Quit before pregnancy	2,838	4,670	60.8%	**0.84 (0.78, 0.90)**	**0.90 (0.84, 0.97)**
Quit after pregnancy	1,590	2,645	60.1%	**0.82 (0.75, 0.89)**	**0.89 (0.81, 0.98)**
Current smoker	503	908	55.4%	**0.67 (0.59, 0.77)**	**0.81 (0.70, 0.94)**
**Alcohol intake during the first trimester**					
Never consumed alcohol	4,363	7,060	61.8%	Reference	Reference
Quit before pregnancy	7,594	12,019	63.2%	1.06 (0.999, 1.13)	1.05 (0.99, 1.12)
Current drinker	1,515	2,305	65.7%	**1.19 (1.07, 1.31)**	**1.16 (1.04, 1.28)**
**NVP prior to 12 gestational weeks**					
Did not experience	2,627	4,049	64.9%	Reference	Reference
Nausea only	5,867	9,249	63.4%	0.94 (0.87, 1.01)	0.98 (0.90, 1.06)
Experienced NVP but could have meals	3,676	5,962	61.7%	**0.87 (0.80, 0.95)**	0.95 (0.87, 1.04)
Experienced NVP and could not have meals	1,302	2,123	61.3%	**0.86 (0.77, 0.96)**	0.93 (0.82, 1.04)
**Stressful events during pregnancy**					
No	7,679	12,113	63.4%	Reference	Reference
Yes	5,793	9,271	62.5%	0.96 (0.91, 1.02)	1.02 (0.96, 1.08)
**Psychological distress during the first trimester (total K6 score)**					
< 5	9,287	14,588	63.7%	Reference	Reference
5–12	3,811	6,103	62.4%	0.95 (0.89, 1.01)	0.96 (0.90, 1.03)
≥ 13	374	693	54.0%	**0.67 (0.58, 0.78)**	**0.74 (0.63, 0.87)**
**MVPA per week**					
< 150 min	11,296	17,434	64.8%	Reference	Reference
≥ 150 min	2,176	3,950	55.1%	**0.67 (0.62, 0.71)**	**0.71 (0.66, 0.76)**
**Dog ownership**					
No	11,842	18,654	63.5%	Reference	Reference
Yes	1,630	2,730	59.7%	**0.85 (0.79, 0.92)**	**0.89 (0.82, 0.97)**
**Physical and mental health (SF-8 score)**					
Physical component summary scores (by point)	-	-	-	**0.99 (0.988, 0.996)**	**0.99 (0.988, 0.996)**
Mental component summary scores (by point)	-	-	-	**1.01 (1.003, 1.010)**	1.00 (0.996, 1.006)

*aOR* adjusted odds ratio, *BMI* body mass index, *CI* confidence interval, *cOR* crude odds ratio, *JPY* Japanese yen, *K6* Kessler 6, *MVPA* moderate to vigorous physical activity, *NVP* nausea and vomiting in pregnancy, *SD* standard deviation, *TV* television

^a^Adjusted for each other; “—” denotes reference

## Discussion

In this study, pregnant Japanese women spent an average of 5.9 h (SD = 3.5) engaged in SB on weekdays during the second/third trimester of pregnancy, and 31.2% of the women were classified in the high-SB group (≥ 8 h/day). With 83,733 participants, this is the largest cohort study to date to examine SB before and after pregnancy. Pre-pregnancy SB for this population was 5.4 h (SD = 3.4), similar to the mean of 5.3 h/day (SD = 3.7) reported in a previous study of 5,346 Japanese adults (including men) [19]. In the present study, 25.6% of the women were classified in the high-SB group (≥ 8 h/day) before pregnancy, which was also similar to the finding (25.3%) in the previous study [19]. As described above, the study population before pregnancy represents the general trend among Japanese adults. Hence, we were able to clearly demonstrate that time spent engaged in SB increases over the course of pregnancy and that the percentage of pregnant women classified in the high-SB group also increases.

A previous study that measured SB by conducting a questionnaire survey and compared the amount of time spent engaged in SB between pregnant and non-pregnant women [23] reported significantly longer durations of SB in pregnant women compared with non-pregnancy. Previous studies using objective measures to assess SB have also shown an increase in SB during pregnancy compared with non-pregnancy [24]. Our results were in line with the results of these previous studies and showed that the mean time spent engaged in SB during pregnancy is longer compared with non-pregnancy. The World Health Organization’s 2020 Guidelines on Physical Activity and Sedentary Behavior stated that there is a lack of evidence regarding the actual amount of time spent engaged in SB among pregnant women [8], and thus the results obtained herein provide important information.

In this study, the amount of time spent engaged in SB was markedly shorter than the 8.6 h reported in a previous study [23], which also involved a questionnaire survey that excluded sleep time. One possible reason for this difference is that the survey in the previous study [23] was conducted as an interview, in contrast to the self-administered questionnaire survey conducted in the present study. However, recent studies have reported significant agreement between responses to the two survey methods [25, 26]. In addition, it has been reported that the higher the minimum daytime temperature, the longer the duration of SB [27]. Given that the previous study was conducted in Singapore, which is located in a tropical region, the amount of time spent engaged in SB may have been longer due to the hotter environment compared with our study region. However, other studies based on objective assessments found that the amount of time spent engaged in SB ranged from 7 to 18 h/day [1, 28–31], although it is difficult to compare our results with those of previous studies because they did not explicitly exclude or clarify sleep time. Previous studies have reported a low correlation between measures of SB obtained through objective assessments and SB assessed using IPAQ [32, 33], so it will be necessary to verify our results by performing objective measures of SB in the future.

The present study identified several factors that were associated with time spent engaged in SB during the second/third trimester of pregnancy. To show the relevant items by pre-pregnancy time spent engaged in SB, we stratified our target population into low-SB (< 8 h/day) and high-SB (≥ 8 h/day) groups before pregnancy. More time spent watching TV and more time spent playing video games were found in the low- and high-SB groups prior to pregnancy (Table 1). Despite these background differences, in both groups stratified by pre-pregnancy hours engaged in SB, more time spent watching TV and more time spent playing video games before pregnancy were associated with ≥ 8 h engaged in SB during pregnancy (Tables 2 and 3). Similarly, the proportion of those who spent ≥ 150 min engaged in MVPA per week and multiparas in either the low- or high-SB group before pregnancy differed (Table 1), but in both groups, spending ≥ 150 min engaged in MVPA per week and multiparas before pregnancy were inversely associated with spending ≥ 8 h engaged in SB during pregnancy (Tables 2 and 3). A previous study has already shown that watching TV during pregnancy is associated with increased time spent engaged in SB [34]. An interesting finding of the present study is that in the pre-pregnancy low-SB group, pre-pregnancy time spent watching TV and time spent playing video games were associated with high SB during pregnancy. It is also important to note that in the pre-pregnancy high-SB group, spending > 150 min engaged in MVPA per week before pregnancy was associated with low SB during pregnancy. Therefore, efforts to reduce time spent watching TV and playing video games and to increase MVPA before pregnancy may prevent the tendency toward high SB during pregnancy.

Parity and some socioeconomic factors were also associated with high SB during pregnancy (Tables 2 and 3). Multiparas are less sedentary compared with primiparas because they spend more time caring for their children, consistent with previous findings [35–37]. From our results, parity and socio-economic factors such as education, household income, and job and residence types were predictive of SB during pregnancy; however, these are not factors that can easily be changed after becoming pregnant. Therefore, it is considered important to find appropriate interventions for each group with these specific factors. As for factors that can more easily be changed after pregnancy, dog ownership was inversely associated with high SB during pregnancy, although only in the pre-pregnancy high-SB group (Table 3). There is evidence that dog ownership increases physical activity [38] and was associated with reduced SB in Japan [39], although it was not associated with MVPA. Our previous study also showed that dog ownership during pregnancy is associated with good postpartum mental health [40]. Although dog ownership may not be an option for everyone, it may be recommended for those who are considering dog ownership, given its potential benefits.

High SF-8 scores for physical and mental health before pregnancy were also inversely associated with high SB during pregnancy (Table 2). High psychological distress and NVP by 12 weeks were also inversely associated with high SB during pregnancy (Table 3). We had expected that severe psychological distress or NVP combined with inadequate nutrition might lead to increased time engaged in SB, but the results were contrary to our expectations. The reasons for this are unknown at this time, but the results are intriguing. At the very least, we can present a positive message to those with NVP or psychological distress in early pregnancy that these conditions are not associated with increased SB in mid-pregnancy.

This study had a number of strengths, including its nationwide coverage and large sample size. Data on the amount of time spent sitting/lying, sleeping, and watching TV were clearly collected; however, these were subjective assessments. There are also several limitations that need to be addressed. Some factors that may have been relevant to the present study (such as health literacy) were not measured. Furthermore, about 13,600 respondents had missing data for the outcome measure; therefore, a selection bias may have been present due to the exclusion of these non-respondents. In addition, because the participants had to retrospectively provide responses, there may have been a recall bias and, thus, estimated times may not have been accurate. The accumulation of further evidence from both objective and subjective evaluations is needed to ascertain the actual amount of time spent engaged in SB during pregnancy.

## Conclusions

Using the results of a nationwide self-administered questionnaire survey in Japan, we examined the actual SB times of women in the second/third trimesters of pregnancy and related factors. The average time spent engaged in SB, excluding sleeping time, was 5.9 h. Factors associated with SB were time spent watching TV, time spent playing video games, engaging in > 150 min of MVPA per week before pregnancy, and some socioeconomic factors. To reduce the amount of time spent engaged in SB, pregnant women need to systematically manage the time they spend watching TV and playing video games and to establish a regular exercise habit before pregnancy.

## Supplementary Information

Supplementary Material 1.

## Data Availability

Data are unsuitable for public deposition due to ethical restrictions and the legal framework of Japan. It is prohibited by the Act on the Protection of Personal Information (Act No. 57 of 30 May 2003, amended on 9 September 2015) to publicly deposit data containing personal information. The Ethical Guidelines for Medical and Health Research Involving Human Subjects enforced by the Japan Ministry of Education, Culture, Sports, Science and Technology and the Ministry of Health, Labour and Welfare also restricts the open sharing of epidemiologic data. All inquiries about access to the data should be sent to jecs-en@nies.go.jp. The person responsible for handling enquiries sent to this e-mail address is Dr. Shoji F. Nakayama, JECS Programme Office, National Institute for Environmental Studies.

## Abbreviations

aOR: adjusted odds ratio
CI: Confidence interval
cOR: crude odds ratio
IPAQ: International Physical Activity Questionnaire
JECS: Japan Environment and Children’s Study
JPY: Japanese yen
K6: Kessler 6
MET: Metabolic equivalent unit
MVPA: Moderate to vigorous physical activity
NVP: Nausea and vomiting during pregnancy
SB: Sedentary behavior
SD: Standard deviation
TV: Television

## References

[1] Hawkins M, Pekow P, Chasan-Taber L. Physical activity, sedentary behavior, and C-reactive protein in pregnancy. Med Sci Sports Exerc. 2014;46(2):284–92. 23860417 10.1249/MSS.0b013e3182a44767

[2] Loprinzi PD, Fitzgerald EM, Woekel E, Cardinal BJ. Association of physical activity and sedentary behavior with biological markers among U.S. pregnant women. J Womens Health (Larchmt). 2013;22(11):953–8. 23968237 10.1089/jwh.2013.4394 PMC3820143

[3] Padmapriya N, Bernard JY, Liang S, Loy SL, Cai S, Zhe IS, et al. Associations of physical activity and sedentary behavior during pregnancy with gestational diabetes mellitus among Asian women in Singapore. BMC Pregnancy Childbirth. 2017;17(1):364. 29047402 10.1186/s12884-017-1537-8 PMC5648496

[4] Yong HY, Mohd Shariff Z, Mohd Yusof BN, Rejali Z, Bindels J, Tee YYS, et al. High physical activity and high sedentary behavior increased the risk of gestational diabetes mellitus among women with excessive gestational weight gain: a prospective study. BMC Pregnancy Childbirth. 2020;20(1):597. 33028258 10.1186/s12884-020-03299-8 PMC7541260

[5] Jones MA, Catov JM, Jeyabalan A, Whitaker KM, Barone GB. Sedentary behaviour and physical activity across pregnancy and birth outcomes. Paediatr Perinat Epidemiol. 2021;35(3):341–9. 33124060 10.1111/ppe.12731 PMC8186559

[6] Reid EW, McNeill JA, Alderdice FA, Tully MA, Holmes VA. Physical activity, sedentary behaviour and fetal macrosomia in uncomplicated pregnancies: a prospective cohort study. Midwifery. 2014;30(12):1202–9. 24861673 10.1016/j.midw.2014.04.010

[7] Pate RR, O’Neill JR, Lobelo F. The evolving definition of “sedentary.” Exerc Sport Sci Rev. 2008;36(4):173–8. 18815485 10.1097/JES.0b013e3181877d1a

[8] Bull FC, Al-Ansari SS, Biddle S, Borodulin K, Buman MP, Cardon G, et al. World Health Organization 2020 guidelines on physical activity and sedentary behaviour. Br J Sports Med. 2020;54(24):1451–62. 33239350 10.1136/bjsports-2020-102955 PMC7719906

[9] Gaston A, Cramp A. Exercise during pregnancy: a review of patterns and determinants. J Sci Med Sport. 2011;14(4):299–305. 21420359 10.1016/j.jsams.2011.02.006

[10] Fazzi C, Saunders DH, Linton K, Norman JE, Reynolds RM. Sedentary behaviours during pregnancy: a systematic review. Int J Behav Nutr Phys Act. 2017;14(1):32. 28298219 10.1186/s12966-017-0485-z PMC5353895

[11] Prince SA, Cardilli L, Reed JL, Saunders TJ, Kite C, Douillette K, et al. A comparison of self-reported and device measured sedentary behaviour in adults: a systematic review and meta-analysis. Int J Behav Nutr Phys Act. 2020;17(1):31. 32131845 10.1186/s12966-020-00938-3 PMC7055033

[12] Craig CL, Marshall AL, Sjostrom M, Bauman AE, Booth ML, Ainsworth BE, et al. International physical activity questionnaire: 12-country reliability and validity. Med Sci Sports Exerc. 2003;35(8):1381–95. 12900694 10.1249/01.MSS.0000078924.61453.FB

[13] Bauman A, Ainsworth BE, Sallis JF, Hagströmer M, Craig CL, Bull FC, et al. The descriptive epidemiology of sitting. A 20-country comparison using the International Physical Activity Questionnaire (IPAQ). Am J Prev Med. 2011;41(2):228–35. 21767731 10.1016/j.amepre.2011.05.003

[14] Kawamoto T, Nitta H, Murata K, Toda E, Tsukamoto N, Hasegawa M, et al. Rationale and study design of the Japan environment and children’s study (JECS). BMC Public Health. 2014;14:25. 24410977 10.1186/1471-2458-14-25 PMC3893509

[15] Michikawa T, Nitta H, Nakayama SF, Yamazaki S, Isobe T, Tamura K, et al. Baseline Profile of Participants in the Japan Environment and Children’s Study (JECS). J Epidemiol. 2018;28(2):99–104. 29093304 10.2188/jea.JE20170018 PMC5792233

[16] Murase N, Katsumura T, Ueda C, Inoue S, Shimomitsu T. Validity and reliability of Japanese version of International Physical Activity Questionnaire. J Health Welf Stat. 2002;49(11):1–9. [Japanese].

[17] Bennie JA, Pedisic Z, van Uffelen JG, Gale J, Banting LK, Vergeer I, et al. The descriptive epidemiology of total physical activity, muscle-strengthening exercises and sedentary behaviour among australian adults–results from the national nutrition and physical activity survey. BMC Public Health. 2016;16:73. 26809451 10.1186/s12889-016-2736-3 PMC4727339

[18] Stamatakis E, Gale J, Bauman A, Ekelund U, Hamer M, Ding D. Sitting time, physical activity, and risk of mortality in adults. J Am Coll Cardiol. 2019;73(16):2062–72. 31023430 10.1016/j.jacc.2019.02.031

[19] Kitayama A, Koohsari MJ, Ishii K, Shibata A, Oka K. Sedentary time in a nationally representative sample of adults in Japan: Prevalence and sociodemographic correlates. Prev Med Rep. 2021;23: 101439. 34178590 10.1016/j.pmedr.2021.101439 PMC8214138

[20] Furukawa TA, Kawakami N, Saitoh M, Ono Y, Nakane Y, Nakamura Y, et al. The performance of the Japanese version of the K6 and K10 in the World Mental Health Survey Japan. Int J Methods Psychiatr Res. 2008;17(3):152–8. 18763695 10.1002/mpr.257 PMC6878390

[21] van Buuren S. Multiple imputation of discrete and continuous data by fully conditional specification. Stat Methods Med Res. 2007;16(3):219–42. 17621469 10.1177/0962280206074463

[22] Rubin DB. Multiple Imputation for Nonresponse in Surveys. New Jersey: Wiley; 2004. https://www.wiley.com/en-gb/Multiple+Imputation+for+Nonresponse+in+Surveys-p-9780471655749.

[23] Padmapriya N, Shen L, Soh SE, Shen Z, Kwek K, Godfrey KM, et al. Physical activity and sedentary behavior patterns before and during pregnancy in a multi-ethnic sample of Asian omen in Singapore. Matern Child Health J. 2015;19(11):2523–35. 26140834 10.1007/s10995-015-1773-3

[24] Gradmark A, Pomeroy J, Renström F, Steiginga S, Persson M, Wright A, et al. Physical activity, sedentary behaviors, and estimated insulin sensitivity and secretion in pregnant and non-pregnant women. BMC Pregnancy Childbirth. 2011;11: 44. 21679399 10.1186/1471-2393-11-44 PMC3130709

[25] Nunes LO, Castanheira ERL, Sanine PR, Akerman M, Nemes MIB. Performance assessment of primary health care facilities in Brazil: Concordance between web-based questionnaire and in-person interviews with health personnel. PLoS ONE. 2023;18(2): e0281085. 36730170 10.1371/journal.pone.0281085 PMC9894387

[26] Gorfinkel L, Stohl M, Shmulewitz D, Hasin D. Self-reported substance use with clinician interviewers versus self-administered surveys. J Stud Alcohol Drugs. 2024;85(1):92–9. 37796626 10.15288/jsad.23-00004 PMC10846601

[27] Ferguson T, Curtis R, Fraysse F, Olds T, Dumuid D, Brown W, et al. Weather associations with physical activity, sedentary behaviour and sleep patterns of Australian adults: a longitudinal study with implications for climate change. Int J Behav Nutr Phys Act. 2023;20(1):30. 36918954 10.1186/s12966-023-01414-4 PMC10012316

[28] Di Fabio DR, Blomme CK, Smith KM, Welk GJ, Campbell CG. Adherence to physical activity guidelines in mid-pregnancy does not reduce sedentary time: an observational study. Int J Behav Nutr Phys Act. 2015;12:27. 25879428 10.1186/s12966-015-0191-7 PMC4345024

[29] Hjorth MF, Kloster S, Girma T, Faurholt-Jepsen D, Andersen G, Kaestel P, et al. Level and intensity of objectively assessed physical activity among pregnant women from urban Ethiopia. BMC Pregnancy Childbirth. 2012;12:154. 23244057 10.1186/1471-2393-12-154 PMC3543321

[30] Evenson KR, Wen F. Prevalence and correlates of objectively measured physical activity and sedentary behavior among US pregnant women. Prev Med. 2011;53(1–2):39–43. 21575654 10.1016/j.ypmed.2011.04.014

[31] Ruifrok AE, Althuizen E, Oostdam N, van Mechelen W, Mol BW, de Groot CJ, et al. The relationship of objectively measured physical activity and sedentary behaviour with gestational weight gain and birth weight. J Pregnancy. 2014;2014:567379. 25309754 10.1155/2014/567379 PMC4189770

[32] Tajima T, Harada K, Oguma Y, Sawada SS. Current status of awareness, knowledge, beliefs, and behavioral intentions regarding the Japanese physical activity guidelines and their relationship with physical activity and sedentary behavior. Nihon Koshu Eisei Zasshi. 2022;69(10):790–804. 35768233 10.11236/jph.21-150

[33] Ishikawa-Takata K, Tabata I, Sasaki S, Rafamantanantsoa HH, Okazaki H, Okubo H, et al. Physical activity level in healthy free-living Japanese estimated by doubly labelled water method and International Physical Activity Questionnaire. Eur J Clin Nutr. 2008;62(7):885–91. 17522602 10.1038/sj.ejcn.1602805

[34] Wagnild JM, Pollard TM. Associations between television time and activPAL-measured duration and pattern of sedentary time among pregnant women at risk of gestational diabetes in the UK. J Phys Act Health. 2020;17(4):471–4. 32035413 10.1123/jpah.2019-0315

[35] Lynch KE, Landsbaugh JR, Whitcomb BW, Pekow P, Markenson G, Chasan-Taber L. Physical activity of pregnant Hispanic women. Am J Prev Med. 2012;43(4):434–9. 22992363 10.1016/j.amepre.2012.06.020 PMC3491652

[36] Izumi M, Manabe E, Kimura S, Iwasa K. Relationship between autonomic nerve activity and physical activity in primiparous and multiparous women. J Japanese Soc Psychosomatic Obstet Gynecol. 2022;27(2):177–84.

[37] Rhodes RE, Blanchard CM, Benoit C, Levy-Milne R, Naylor PJ, Symons Downs D, et al. Physical activity and sedentary behavior across 12 months in cohort samples of couples without children, expecting their first child, and expecting their second child. J Behav Med. 2014;37(3):533–42. 23606310 10.1007/s10865-013-9508-7

[38] Christian HE, Westgarth C, Bauman A, Richards EA, Rhodes RE, Evenson KR, et al. Dog ownership and physical activity: a review of the evidence. J Phys Act Health. 2013;10(5):750–9. 23006510 10.1123/jpah.10.5.750

[39] Koohsari MJ, Shibata A, Ishii K, Kurosawa S, Yasunaga A, Hanibuchi T, et al. Dog ownership and adults’ objectively-assessed sedentary behaviour and physical activity. Sci Rep. 2020;10(1):17487. 33060697 10.1038/s41598-020-74365-6 PMC7562738

[40] Matsumura K, Hamazaki K, Tsuchida A, Inadera H. Pet ownership during pregnancy and mothers’ mental health conditions up to 1 year postpartum: a nationwide birth cohort-the Japan environment and children’s study. Soc Sci Med. 2022;309:115216. 36029711 10.1016/j.socscimed.2022.115216

