# Supplementary material for: Factors related to sedentary behavior of pregnant women during the second/third trimester: prospective results from the large-scale Japan Environment and Children’s Study
Source: BMC Public Health. 2024 Nov 15;24:3182. doi: 10.1186/s12889-024-20574-x (PMC11566995; doi:10.1186/s12889-024-20574-x)
Supplement: Supplementary file 1 — Supplementary Material 1. [file 12889_2024_20574_MOESM1_ESM.docx]

Supplementary Information for

Factors related to sedentary behavior of pregnancy women during the second/third trimester: prospective results from the large-scale Japan Environment and Children’s Study

Makie Nagai, Akiko Tsuchida, Kenta Matsumura, Haruka Kasamatsu, Hidekuni Inadera,
and the Japan Environment and Children’s Study Group

**
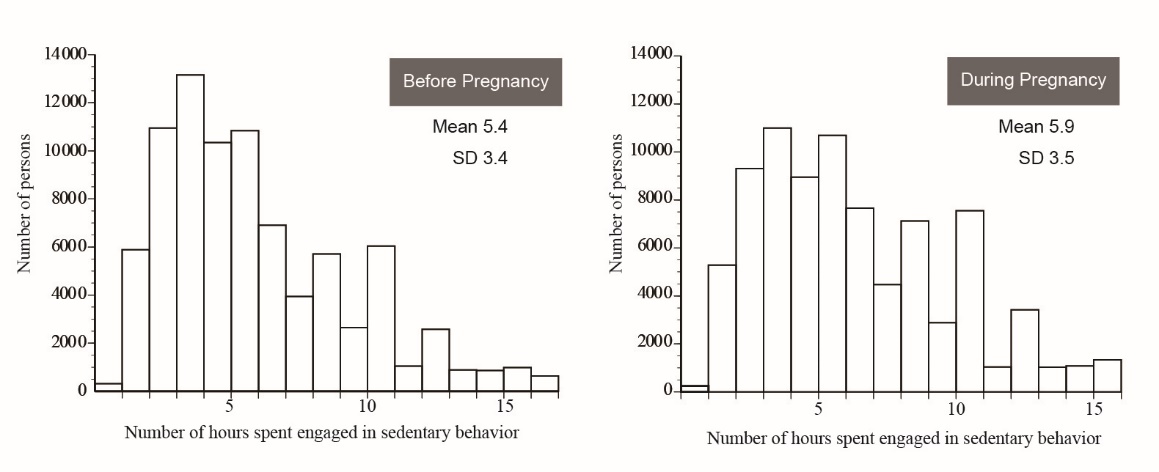
**

**Supplementary Figure 1.** Distribution of number of hours spent engaged in sedentary behavior (N=83,733).
